# Host depletion kits improve microbiome analyses in environmental samples: seagrass as a test case

**DOI:** 10.1093/ismeco/ycag082

**Published:** 2026-03-28

**Authors:** Philipp Schmelz, Stefan Eckensperger, Jay Osvatic, Joana Séneca, Hanin Alzubaidy, Jillian M Petersen

**Affiliations:** Centre for Microbiology and Environmental Systems Science, University of Vienna, Vienna 1030, Austria; Centre for Microbiology and Environmental Systems Science, University of Vienna, Vienna 1030, Austria; River Ecosystems Laboratory, Alpine and Polar Environmental Research Centre, School of Architecture, Civil and Environmental Engineering, École Polytechnique Fédérale de Lausanne, Sion 1950, Switzerland; Joint Microbiome Facility of the Medical University of Vienna and the University of Vienna, Vienna 1030, Austria; Department of Laboratory Medicine, Medical University of Vienna, Vienna 1090, Austria; Centre for Microbiology and Environmental Systems Science, University of Vienna, Vienna 1030, Austria; Joint Microbiome Facility of the Medical University of Vienna and the University of Vienna, Vienna 1030, Austria; Department of Laboratory Medicine, Medical University of Vienna, Vienna 1090, Austria; Centre for Microbiology and Environmental Systems Science, University of Vienna, Vienna 1030, Austria; Marine Evolutionary Ecology, GEOMAR Helmholtz-Center for Ocean Research Kiel, Kiel 24148, Germany; Centre for Microbiology and Environmental Systems Science, University of Vienna, Vienna 1030, Austria; Vienna Doctoral School for Microbiology and Environmental Science, University of Vienna, Vienna 1030, Austria; Environment and Climate Hub, University of Vienna, Vienna 1090, Austria

**Keywords:** microbiome, DNA extraction, environmental samples, seagrass, Sedimenticolaceae

## Abstract

All plants and animals associate with specific communities of symbiotic microorganisms. Characterizing the diversity and functions of these communities is essential for understanding their roles in host health; however, such efforts are often hindered by the dominance of host-derived material in, e.g. DNA extractions. Although various commercial host DNA depletion kits have been developed to overcome these challenges, they have not yet been systematically tested on environmental samples. We used *Zostera marina*, globally the most widespread seagrass species, as a test case to assess the effectiveness of three different commercially available host DNA depletion kits: QIAamp DNA Microbiome Kit, HostZero Microbial Enrichment Kit, and NEBNext Microbiome DNA Enrichment Kit, when compared to the widely used DNeasy PowerSoil Pro Kit. All three host depletion kits substantially reduced the relative proportion of host DNA, as assessed by 16S rRNA gene amplicon sequencing, and enriched previously identified seagrass-associated bacteria. Furthermore, in metagenomes, only samples processed with host depletion methods allowed for the assembly of metagenome-assembled genomes with high completeness and low contamination. Metagenomic analysis further enabled the recovery of seagrass root core microbiome members, including previously undetected members of the family Sedimenticolaceae, highlighting the value of these techniques for uncovering novel host-associated microbial diversity in environmental samples such as marine plants.

## Introduction

Metagenome sequencing has become indispensable in microbial ecology, revealing which microorganisms interact with each other, with plant, animal, or protist hosts or in novel environments, and the potential functions underpinning these relationships [1, 2]. Many studies have already investigated the microbiomes of diverse hosts, from mammals [3] to plants [4]. A major limitation of these sequencing methods, however, is the overwhelming presence of host DNA, which tends to dominate metagenome sequence reads. A notorious example stems from the Human Microbiome Project [2], in which >90% of the reads from different tissue samples aligned to the human genome, hindering in particular the analysis of low-abundance microbial taxa.

To tackle this issue, several studies have compared DNA extraction protocols to reduce the presence of host DNA in samples such as human feces, saliva, and infected tissues [2, 5–7]. These protocols often include a host DNA depletion step either before (“pre-extraction”) or after (“post-extraction”) nucleic acid extractions. Commercially available kits based on pre-extraction approaches include the HostZero Microbial DNA kit (Zymo Research) and the QIAamp DNA Microbiome kit (Qiagen), which enzymatically degrade eukaryotic DNA prior to extraction. In contrast, the post-extraction NEBNext Microbiome DNA Enrichment kit (New England Biolabs) uses magnetic beads that selectively bind characteristic eukaryotic methylations to remove host DNA after extraction. Although several studies have investigated microbial communities using at least one of these three kits [5, 8–11], few studies have systematically compared them for microbial profiling [5, 8], and none have evaluated their efficacy in seagrass or other marine plant samples.

Understanding the importance of seagrasses and their associated microbiomes has become a research priority in marine and microbial sciences. Seagrasses are marine flowering plants that recolonized the ocean several times independently during their evolution [12]. They play crucial roles in maintaining ecosystem functions, providing protection and food for other organisms, and serving as a large carbon sink with hundreds of tons of organic carbon stored per hectare through photosynthesis and carbon fixation [13, 14]. Anthropogenic disturbances such as rising sea temperatures and eutrophication of marine ecosystems therefore have the potential to disrupt seagrass ecosystems [15, 16]. As such, the past few years have seen large-scale attempts to restore these habitats, with efforts already demonstrating partial success, such as in the recovery of a *Zostera marina* meadow [17]. Additionally, growing attention has turned to the role of seagrasses and their associated microbiomes in ecosystem resilience and recovery.

The seagrass microbiome, like other plant microbiomes, can be broadly divided into an above-ground compartment, the phyllosphere, and a below-ground compartment, the rhizosphere [4, 13, 18]. The phyllosphere is primarily colonized by heterotrophic epiphytic organisms from the surrounding seawater, while the rhizosphere hosts highly diverse bacterial communities that are taxonomically distinct from those in surrounding sediments [13]. In root microbiomes, the local environment may exert a stronger influence on bacterial community composition than host species identity [19]. Nevertheless, seagrass roots have been shown to harbor “core” microbiomes—bacterial communities consistently associated with seagrasses across diverse geographic locations. These core taxa include classes such as α- and γ-proteobacteria, formerly classified “δ”- and “ε”-proteobacteria (now reclassified [20, 21]), Bacteroidetes, and Clostridiales [13, 19, 22–29]. They may serve as indicators of seagrass health and stress [28] and contribute to key biogeochemical processes such as sulfur cycling by sulfate-reducing “δ”-proteobacteria and sulfur-oxidizing γ- or “ε”-proteobacteria [20, 30, 31].

For the identification of seagrass-associated bacteria, 16S rRNA gene amplicon and metagenomic surveys are commonly employed [22, 23, 32–35]. Similar to human tissue samples [2], the dominance of plant host DNA in these samples impairs the detection of their associated microbiomes. For example, a metagenomic survey of *Z. marina* rhizospheres yielded 20 draft genomes, but only 4 of them had a completeness >70% and contamination <5% [23], representing only a small fraction of the detectable microbial diversity. However, other studies have more frequently used sediment samples from seagrass meadows, instead of the actual plant tissues [36, 37]. This approach might be suitable to gain insights into the overall microbial community composition of seagrasses but potentially misses out on specialized bacteria living within or on plant tissues.

In this study, we analyzed microbiomes of seagrass root samples using three host DNA depletion kits (HostZero, QIAamp, and NEBNext), including both standard and custom-modified protocols. Additionally, we compared their effectiveness against the commonly used DNeasy PowerSoil Pro Kit (Qiagen) in reducing host DNA contamination and enhancing microbial detection, as proof of principle for the potential use of host depletion methods in future seagrass and possibly other plant microbiome studies.

## Materials and methods

### Sampling and sample preparation

*Zostera marina* was sampled in Ferrol, Spain, by SCUBA divers in a subtidal area 5 m below sea level in October 2023 (43.27.51.0 N; 8.17.02.5 W). A 20 m × 30 m area was marked out as the sampling site (Suppl. S1). Within this area, 25 samples were collected, each containing the rhizome with attached roots and blades. Five plants were sampled from each of three smaller 2 m × 3 m quadrants, spaced 3 m apart (Suppl. S1). The remaining 10 samples were dispersedly collected across the large quadrant. Sample preparation was carried out on the same day as sampling. Samples were dissected into blades and root + rhizome pieces. Dissecting tools and working surfaces were cleaned with 70% ethanol between each plant. Sediment was removed from samples using autoclaved Milli-Q water. The dissected root + rhizome and the outermost leaves were placed in 2 ml Eppendorf tubes, snap-frozen in liquid nitrogen, and stored at −75°C. Before nucleic acid extractions, samples were homogenized using autoclaved mortars and pestles in liquid nitrogen until finely powdered. Gloves, autoclaved pincers, and spatulas were changed between samples, and surfaces were cleaned with 70% ethanol. Aliquots (~200 mg) were prepared and either stored at −75°C or used immediately.

### DNA extractions

Preliminary tests were conducted to evaluate host DNA depletion kits and to optimize protocols. Based on these results, four kits and their protocol modifications were selected for DNA extraction of ~200 mg from 24 *Z. marina* root + rhizome samples (Suppl. Protocols, Suppl. S2). We tested two pre-extraction host depletion kits: QIAamp DNA Microbiome Kit (Qiagen, Cat. No. 51704) and the HostZERO Microbial DNA Kit (Zymo Research, Cat. No. D4310). Modifications were made to both kits due to the liquid sample requirement. In addition, we tested a post-extraction host-depleted method, which included DNA extraction with the Wizard HMW DNA Extraction Kit (Promega, Cat. No. A2920), followed by the NEBNext Microbiome DNA Enrichment Kit (New England Biolabs, Cat. No. E2612S/L) to selectively remove eukaryotic CpG-methylated DNA via magnetic beads. For both kits, the manufacturer’s instructions were followed. As a control, the DNeasy PowerSoil Pro Kit (Qiagen, Cat. No. 47014) was used to extract total DNA, including host and associated microbiota, according to the manufacturer’s instructions. These samples served as a “baseline” for comparison with host DNA depletion kits. We tested potential biases introduced via host depletion methods using components and reagents from all kits and 75 μl of a mock community (ZymoBIOMICS, Cat. No. D6300, Suppl. SE) as a positive or Milli-Q water as a negative control. DNA concentrations were measured using Qubit 4 (Thermo Fisher Scientific) with the Qubit dsDNA HS Assay Kit (Thermo Fisher Scientific, Cat. No. Q32851).

### Sequencing and data analysis

#### Amplicon sequencing

Sequencing and raw data processing were performed at the Joint Microbiome Facility (University of Vienna and Medical University of Vienna). Sequencing of seagrass samples was processed under the sequencing ID JMF-2404-07, and mock communities under JMF-2502-10. Using a previously described two-step PCR protocol [38], the V4 hypervariable region of the bacterial and archaeal 16S rRNA gene was amplified using headed primers 515F (5′-GTGYCAGCMGCCGCGGTAA) [39] and 806R (5′-GGACTACNVGGGTWTCTAAT) [40] using the following PCR protocol: initial denaturation 94°C for 3 min; 30 cycles of: denaturation 94°C for 45 s, annealing 52°C for 60 s, elongation 72°C for 90 s, and final elongation 72°C for 10 min. The first step PCR product was subsequently targeted on a second PCR where unique dual barcodes (UDB-12) were introduced and amplified as follows: 94°C for 4 min; seven cycles of: 94°C for 30 s, 52°C for 30 s, 72°C for 60 s, and 72°C for 7 min [38]. Library preparation was performed as previously described [38], using the innuPREP PCRpure Kit (Analytik Jena) for multiplexing, and the TruSeq Nano DNA Library Prep Kit (Illumina) according to the manufacturer’s instructions but skipping the fragmentation step. Libraries were sequenced on an Illumina MiSeq using the MiSeq Reagent kit v3 (2 × 300 cycle paired-end, Illumina) [38]. Amplicon pools were extracted from the raw sequencing data using the FASTQ workflow in BaseSpace (Illumina) with default parameters. Raw data processing was performed as described previously [38]. Demultiplexing was performed with the Python package demultiplex [41], allowing one mismatch for barcodes and two mismatches for linkers and primers. Amplicon sequence variants (ASVs) were inferred using the DADA2 R package v1.42 [42] applying the recommended workflow [43]. FASTQ reads 1 and 2 were trimmed at 220 and 150 nts with allowed expected errors of 2. ASV sequences were subsequently classified using DADA2 and the SILVA database SSU Ref NR 99 release 138.1 [44, 45] using a confidence threshold of 0.5. Statistical analyses were performed in R (v4.2.2) [46] using packages including phyloseq (v1.42.0) [47], microViz (v0.12.1) [48], dplyr (v1.1.4) [49], ggplot2 (v3.4.4) [50], vegan (v2.6.4) [51], patchwork (v1.2.0) [52], tidyverse (v2.0.0) [53], microbiomeutilities (v1.0.17) [54], FSA (v0.10.0) [55], VennDiagram (v1.7.0) [56], usedist (v0.4.0) [57], ecole (v0.9.2021) [58], and DESeq2 (v1.38.3) [59].

**Figure 1:**
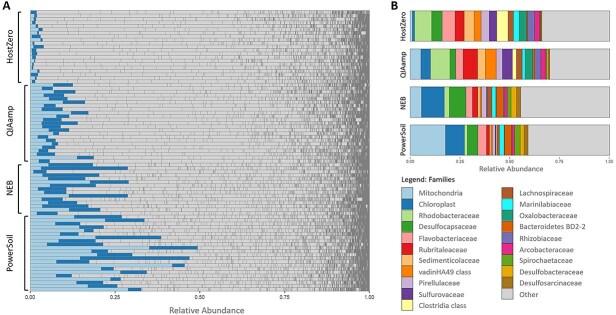
Microbial composition*.* (A) Relative abundance of host contents for each individual sample. (B) Grouped microbial distribution for each kit. Colors represent 20 most abundant taxonomically assigned families.

The average sequencing depth of *Z. marina* root + rhizome samples was 12 748 reads/sample, with a total number of 10 269 ASVs detected. Samples were rarified to 9000 reads to compare different sample sizes (seed = 123). We evaluated host depletion efficiency for all remaining samples (*n* = 81, Fig. 1, Suppl. S2).

#### Metagenomic sequencing

A selection of five samples per kit (total = 20), based on the highest DNA concentrations obtained by pre-extraction kits (0.2–0.8 ng/μl), was selected for metagenomic sequencing (Sequencing ID JMF-2404-07; Suppl. S2). Libraries were prepared for sequencing using the NEBNext Ultra II FS DNA Library Prep Kit for Illumina according to the manufacturer’s instructions, pooled equimolarly, and sequenced on an Illumina NovaSeq 6000 (2 × 100 bp, 650 million reads, 1 S Prime flowcell, 200 cycles). A quality-control snakemake v8.19.3 [60] workflow was used to trim read libraries based on quality scores. Briefly, the read libraries were quality-checked using fastQC v0.12.1 [61] and quality statistics were merged using multiQC v1.21 [62]. Adapters were trimmed, and phiX contamination was removed using BBDuk (part of BBMap v39.06 [63]) to a minimum of 50 bp in length. Reads were *k*-trimmed from the right with a kmer of 21, a minimum *k*mer of 11, and a hamming distance of two, along with the “tpe” and “tbo” options. Quality trimming was performed from the right with a Q-score of 15. BBMap’s reformat.sh was used to interleave the read library.

Libraries were assembled using SPAdes v4.0.0 [64] with *k*mers of 21–121 in steps of 10 and the “isolate” mode. Contigs under 1000 bp were removed from the resulting assembly using reformat.sh of BBMap. Metabat v2.15 [65] was used to generate putative metagenome-assembled genomes (MAGs) without coverage information using a minimum length of 1500 bp (minimum possible length). Coverage information for the assembly was generated with BBMap with a minimum identity of 98% using the read library. The resulting bam file was sorted using samtools v1.20 [66]. A depth file was generated using jgi_summarize_bam_contig_depths (Metabat), and the assembly was binned into putative MAGs again using the depth file in Metabat. All putative MAGs from the assembly were dereplicated, along with the assembly, using dREP v3.5.0 [67]. Taxonomy of dereplicated MAGs was determined using gtdbtk v2.4.0’s [68] classify_wf with database r220, skipping average nucleotide identity (ANI) screening.

Ribosomal RNA sequences were generated from the non-interleaved read libraries using phyloflash v3.4.2 [69] with the “almosteverything” option and the SILVA v138.1 [45] database. Alignments from GTDB-Tk were used to generate a phylogeny in IQtree v2.3.6 [70], with model testing, ultrafast bootstrap, and 1000 replicates. MAG completeness, contamination, and strain heterogeneity were evaluated using CheckM v1.2.0 [71] and checked for their metabolic potential in Rapid Annotation using Subsystem Technology (RAST) using the RASTtk pipeline [72]. Relative abundance and coverage for the MAGs were calculated using coverM v0.7.0 [73] for all read libraries. Shotgun metagenomic reads were aligned to the *Z. marina* reference genome [74] at 95% identity using BBMap v39.06, to visualize the percentage of *Z. marina* and non-*Z. marina* reads. The reads matching the references were counted to determine the amount of host contamination left in the metagenome (Fig. 3B). BBMap v39.06 was also used to determine the percent mapped from each assembly or MAG to all readsets.

## Results

### Pre-extraction kits most effectively reduced host-derived 16S rRNA gene reads

The control kit PowerSoil yielded the highest average DNA concentrations (25.5 ng/μl), followed by the post-extraction method New England Biolabs (NEB; 15.4 ng/μl), and lastly, the pre-extraction methods QIAamp and HostZero with low but sufficient concentrations for sequencing (<1 ng/μl) (Suppl. S2). Rarefaction of the sequencing data led to the exclusion of three PowerSoil, eight NEB, one QIAamp, and three HostZero samples (containing <9000 reads) from the analysis (Suppl. S3). We assessed the proportion of host DNA in extracted samples by comparing the relative abundance of mitochondrial and chloroplast 16S rRNA gene amplicon sequences across the different extraction kits. Out of all kits, PowerSoil yielded the highest proportion of host reads (range: 12.19%–49.33%, average: 27.18%), followed by NEB (range: 4.74%–29.96%, average: 17.03%), QIAamp (range: 5.22%–18.64%, average: 10.06%), and HostZero (range: 0.82%–3.94%, average: 2.07%). Compared to PowerSoil, all host depletion kits successfully reduced mitochondrial read counts, with HostZero resulting in an almost complete depletion. Chloroplasts, however, were only reduced by QIAamp and again almost completely depleted by HostZero (Fig. 1).

### Distinct microbial community structure between PowerSoil and NEB-extracted samples compared to QIAamp and HostZero

**Figure 2:**
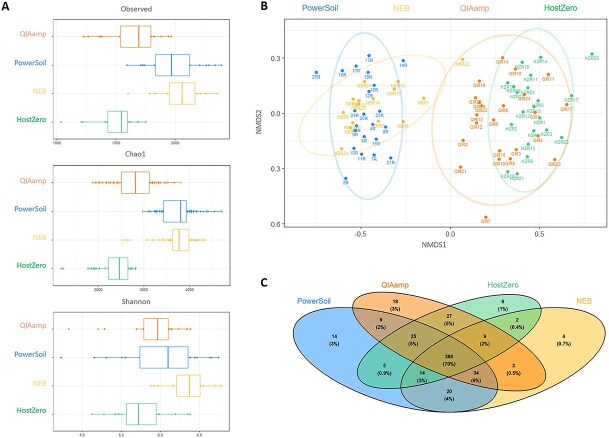
Extraction methods influence community composition. (A) Alpha diversity measurements for Observed, Chao1 richness estimator, and Shannon diversity index across the tested extraction kits. (B) Beta diversity based on a Bray–Curtis distance matrix. Stress: 0.128. (C) Unique and shared microbial families across tested extraction kits.

NEB and PowerSoil extracted samples yielded higher species richness (Observed, Chao1) and diversity (Shannon) estimates, compared to pre-extraction kits QIAamp and HostZero. We used a nonparametric Kruskal–Wallis test with Benjamini–Hochberg adjustment to check for significant differences in alpha diversity. No significant differences of variables were found between PowerSoil and NEB in Observed and Chao1 (*P* > .05), between QIAamp and PowerSoil in Shannon (*P* > .05), and between QIAamp and HostZero in all three metrics (*P* > .05) (Fig. 2A, Suppl. S4). Beta diversity analysis using nonmetric multidimensional scaling (NMDS) with a Bray–Curtis distance matrix showed that NEB and PowerSoil extracted samples clustered together, whereas QIAamp and HostZero formed distinct clusters (Fig. 2B). We then used pairwise PERMANOVA to reveal differences in microbial community structure between treatments after confirming that dispersions from centroids do not differ significantly using permutation-based test of group dispersions (*P* > .05, permutations = 99, Suppl. S5). According to PERMANOVA, all treatments contained significantly different taxonomic families (*P* < .05 (0.0001); permutations = 9999; *R*^2^ = PowerSoil vs NEB: 0.08, QIAamp vs HostZero: 0.1, others: 0.25–0.31; Suppl. S5). To evaluate bacterial community compositional differences, we analyzed samples with representatives across all four treatments after rarefaction (*n* = 11 per treatment, Suppl. S2) and removed chloroplast and mitochondrial ASVs. Here, a pairwise PERMANOVA test (permutations = 9999) revealed that microbial community structure showed no significant differences between PowerSoil and NEB extractions (*P* > .05, *R*^2^ = 0.06), whereas all other community comparisons were significantly different (*P* < .05 (0.001–0.0001); *R*^2^ = QIAamp vs HostZero: 0.09, others: 0.25–0.29; Suppl. S5). We also compared each sample at the family level. QIAamp-extracted samples had the highest number of families (485), followed by PowerSoil (481), HostZero (448), and NEB (446). All kits shared 360 families, accounting for 70% of total microbial diversity in the dataset. The remaining families were unique or variably shared among treatments. Host depletion kits collectively lacked 121 families compared to PowerSoil: 53 were missing in NEB and QIAamp, and 77 in HostZero. Most missing taxa had low relative abundance even in PowerSoil, and more abundant taxa mostly overlapped with NEB. Conversely, NEB had 18 unique families compared to PowerSoil, QIAamp 57, and HostZero 44 (Fig. 2C).

We used DESeq2 to identify significantly differentially abundant ASVs between the four extraction methods (*a* = 0.05). PowerSoil and NEB, respectively, enriched more taxa overall than pre-extraction kits. However, enriched taxa in both QIAamp and HostZero were commonly found in other studies of the seagrass root core microbiome (α-Proteobacteria: Rhizobiaceae, Rhodobacteraceae, Sphingomonadaceae; γ-Proteobacteria: Sedimenticolaceae; “δ”-Proteobacteria: Desulfarculaceae, Dusulfatiglandaceae, Desulfocapsaceae, Desulfomonilaceae; Campylobacterota: Sulfurovaceae; Clostridiales: Christensenellaceae), whereas PowerSoil and NEB additionally enriched taxa beyond the core microbiome (Suppl. S6). As PowerSoil and NEB extracted samples differed mostly in mitochondrial reads, we again only analyzed samples with representatives across all four treatments (*n* = 11 per treatment, Suppl. S2) without mitochondrial and chloroplast DNA. Compositional differences between each kit combination remained, except that using this approach, there were no ASVs significantly differentially abundant between NEB and PowerSoil-extractions (*P* > .05, Suppl. S7).

### Host representation was lowest in metagenomes using pre-extraction kits

**Figure 3:**
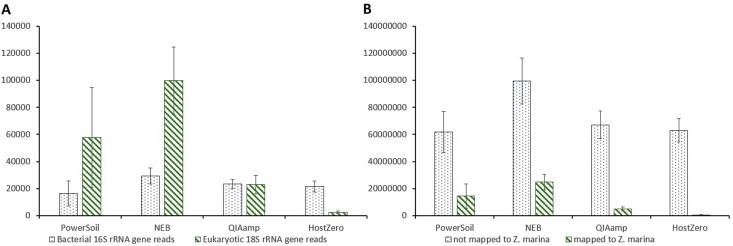
Pre-extraction kits highly reduce host-associated reads. (A) Metagenomic reads mapped to the SILVA SSU rRNA database. (B) Metagenomic reads mapped to the *Z. marina* reference genome at 95% identity, or to everything but *Z. marina*. Error bars represent the standard deviation for the samples.

We evaluated metagenomic reads aligned to the SILVA small subunit (SSU) rRNA database using Phyloflash [69]. NEB extractions yielded the highest average number of host-associated 18S rRNA reads, followed by PowerSoil, QIAamp, and HostZero (Fig. 3A, Suppl. S8). As expected, most eukaryotic reads belonged to the Archaeplastida supergroup, primarily *Z. marina*, with others in the Amorphea, Stramenopiles/Alveolates/Rhizarians supergroup and unidentified groups. All depletion kits successfully increased bacterial 16S rRNA gene reads: 90% of reads in HostZero and 51% in QIAamp mapped to bacteria, compared to <25% in NEB and PowerSoil (Fig. 3A, Suppl. S8). Alignment of metagenomic reads to the *Z. marina* reference genome [74], and *Z. nigricaulis*, the only other publicly available *Zostera* genome-assembly on NCBI, mirrored SSU rRNA patterns (Suppl. S9 and S10): HostZero and QIAamp had the highest proportion of total bacterial reads, followed by PowerSoil and NEB. On average, 0.8% of reads in HostZero, 6.9% in QIAamp, 19% in PowerSoil, and 19.9% in NEB aligned to the host genome (Fig. 3B, Suppl. S9), of which only HostZero achieved a significant reduction of host-associated reads compared to PowerSoil and NEB (*P* < .05, Suppl. S11).

### High-quality metagenome-assembled genome recovery requires host depletion kits

**Figure 4:**
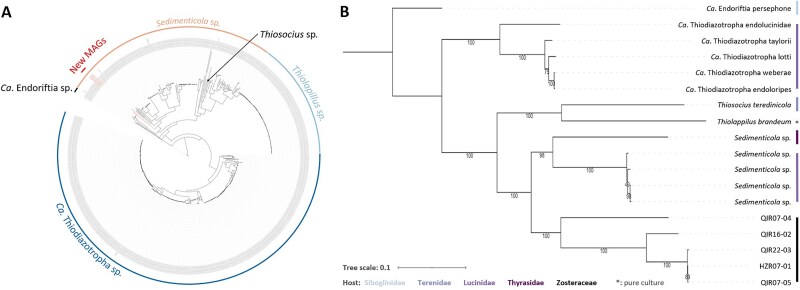
Phylogenetic trees of Sedimenticolaceae. (A) Phylogenetic tree of newly assembled Sedimenticolaceae MAGs, depicted in red, against all their relatives. (B) shows these new MAGs against selected representative species of main genera from Sedimenticolaceae with complete genomes or good assembly levels (Suppl. S14). Host colors refer to the legend at the bottom, and indicate the host group the MAG stems from. Bootstrap values are indicated below each leaf.

We constructed MAGs using a 75% completeness cutoff. Only host depletion kits generated MAGs exceeding this threshold, with completeness ranging from 75% to 99.9% (Suppl. S12 and S13). They represented between 10$\times$ (taxon of the family Vallitaleaceae, relative abundance: 0.45%) to 386$\times$ (taxon of the family Sedimenticolaceae, relative abundance: 19.25%) genomic coverage (Suppl. S15 and S16). Metagenomes from HostZero extractions resulted in the assembly of 31 MAGs, QIAamp in 27, and NEB in 19. In total, the 77 MAGs represented 32 taxonomic families, most of which represented taxonomic novelties based on the placement in the Genome Taxonomy Database (GTDB) reference tree, using ANI and relative evolutionary divergence (RED) [68, 75]. These included 9 families in NEB extractions, 1 in QIAamp, 8 in HostZero, 12 shared between QIAamp and HostZero, and 2 common to all three kits. Of these, 27 families were associated with the seagrass root core microbiome in previous studies (Suppl. S12). For example, the family Sedimenticolaceae, known to contain sulfur-oxidizing bacteria, was recovered only with pre-extraction kits. Sedimenticolaceae MAGs showed 75%–87% completeness and 2%–4% contamination (Suppl. S13). Phylogenetic analysis with all publicly available Sedimenticolaceae genomes from NCBI showed that the assembled MAGs formed distinct clades within the *Sedimenticola* genus (Fig. 4). Three MAGs (QIR22-03, HZR07-01, and QIR07-05) clustered closely, while two others (QIR07-04 and QIR16-02) formed separate branches (Fig. 4). All assembled genomes were, according to RAST [72], 1.8–2.2 Mbps, smaller than the reference genomes (Suppl. S14).

### Host depletion drives assembly success rather than loss of microbial diversity representation

**Figure 5:**
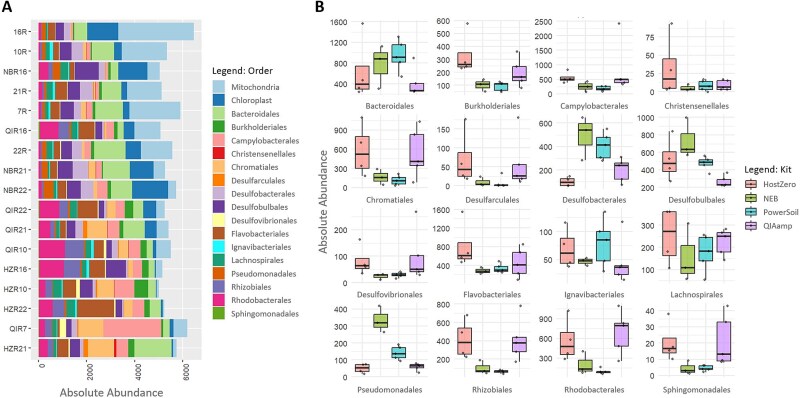
Order abundances in amplicon data of obtained MAGs. (A) Absolute abundances for each individual sample. Colors represent taxonomically assigned orders. (B) Boxplot for absolute abundances. Dots represent individual samples.

We traced the metagenomic data back to the rarefied amplicon data for taxa, resulting in high-quality MAGs. As ASVs do not always correspond to the taxonomically assigned MAG, we grouped them together at the order taxonomic level for all core microbiome members (excluding the XYA12-FULL-58-9 genus, Myxococcota phylum). With the exception of Desulfobulbales (“δ”-Proteobacteria), all the genome assemblies obtained already had the highest absolute abundances for host-depleted methods in the amplicon data. Conversely, Powersoil yielded comparably high read counts to those of host depletion kits, resulting in MAGs for orders including Bacteroidales, Desulfobacterales, and Ignavibacterales. However, it did not generate high-quality assemblies, presumably due to the higher presence of host DNA (Fig. 5).

To determine whether the recovery of high-quality MAGs was due to host depletion rather than reduced microbial diversity, we aligned all reads to assembled contigs and compared mappings across treatments (Suppl. S17). When reads from one treatment show high mapping percentages to their own assemblies, we consider all species within the sample to be well represented. When the same reads show high mapping percentages to assemblies from different treatments, all species within these samples are well represented. Pre-extraction method reads showed the highest alignment percentage to their own assemblies and comparable mappings to assemblies from PowerSoil and NEB (Table 1). Despite all percentages being very low due to the environmental origin of the samples, if loss of diversity were the main reason for assembly improvement, mappings to the other assemblies would be much lower. This suggests that in pre-extraction kits, host depletion, rather than loss of microbial content, is the driving factor that enables successful MAG recovery, even if some microbial DNA likely did not withstand the enzymatic treatment. Nevertheless, matching read percentages are no indicators for similar family occurrences (MAGs) but rather support the idea that reads of one treatment would be as successful in creating assemblies as reads from the other treatment.

**Table 1:** Percentage of reads mapped from each assembly to all read sets, averaging all samples by each treatment.

Assemblies	PowerSoil reads	NEB reads	QIAamp reads	HostZero reads
PowerSoil	2.37%	1.99%	2.07%	1.75%
NEB	3.95%	6.2%	6.01%	5.1%
QIAamp	2.48%	3.06%	9.85%	13.23%
HostZero	1.5%	1.64%	8.31%	13.38%

## Discussion

All the host depletion kits tested in this study successfully reduced host DNA content and enabled a deeper analysis of the seagrass root core microbiome. Compared to PowerSoil, the NEB kit reduced the abundance of both chloroplast and mitochondrial ASVs by 37.3% (Fig. 1). While it yielded overall fewer taxonomic groups at the family level (Fig. 2C), NEB showed higher average alpha diversity than the control, suggesting greater representation of species within the detected families (Fig. 2A). Additionally, NEB-treated samples were not significantly different from PowerSoil in community composition based on pairwise PERMANOVA (Fig. 2B) or differential taxonomic abundance (DESeq2), indicating that NEB may serve as a viable alternative to PowerSoil, and may be comparable to the wealth of studies available based on this widely used kit in environmental microbiology research, as it retains the bulk microbial community structure while reducing host DNA content in the sample types tested. However, metagenomic data from NEB showed the highest number of reads mapped to both bacterial and eukaryotic SSU rRNA genes, even surpassing PowerSoil (Fig. 3A). This could be attributed to the use of the Promega Wizard HMW kit prior to NEB treatment, which may have improved DNA quality and thereby enhanced sequencing efficiency. NEB samples not only generated a larger number of paired-end reads but also lost fewer reads after initial filtering compared to the other extraction kits, increasing total metagenomic coverage (Suppl. S8 and S18). Another possible reason for the high host DNA levels in NEB samples is the limited specificity of CpG-methylation-based removal in plants. Unlike animals, which primarily exhibit CpG methylation, plants also utilize CHG and CHH methylation patterns (H = A, C, or T) [76, 77], potentially reducing the efficiency of host DNA depletion with NEB.

Our metagenomes had a large proportion of “unmapped” reads, which were not bacterial but did not map to the available *Z. marina* genome, even with relaxed mapping settings. This was consistent across all treatments including PowerSoil (Fig. 3B). This may be due to our sample material not being fully representative of the subspecies used to generate the reference *Z. marina* genome [74]. Further investigating the metagenomic reads of the host may reveal unexpected diversity within this species.

The pre-extraction kits HostZero and QIAamp achieved the highest host DNA depletion, reducing host-derived reads by over 90% and 60%, respectively, in the amplicon dataset (Fig. 1). Both kits also resulted in significantly different microbial compositions compared with PowerSoil and exhibited lower overall diversity (Fig. 2). This raises the question of whether enzymes implemented in pre-extraction kits selectively target eukaryotic cells or may also affect specific microbial groups. Previous studies have shown that host depletion methods can be biased against gram-negative bacteria in human samples [78, 79], as did our results in the mock community experiment (Suppl. SE). We did not observe this pattern in our seagrass data, possibly due to the predominance of gram-negative taxa in seagrass root microbiomes. However, pre-extraction kits also enriched gram-positive bacteria such as Clostridiales in our amplicon-based analyses. In metagenomic analyses, QIAamp and especially HostZero further demonstrated superior host DNA depletion and microbial enrichment compared with PowerSoil and NEB (Fig. 3).

In our study, only host depletion kits enabled the recovery of MAGs with >75% completeness. Most of the MAGs belonged to known seagrass core microbiome families (Suppl. S12). Pre-extraction kits primarily enriched and recovered MAGs from α-proteobacteria and Clostridiales, while both pre- and post-extraction kits captured additional core members within γ-, δ-, and ε-Proteobacteria and Bacteroidetes. Interestingly, NEB and the pre-extraction kits yielded largely nonoverlapping MAGs, with few exceptions such as an unidentified member within Rhizobiales and *Saccharicrinis*. Within the phylum Desulfobacterota (formerly δ-proteobacteria), NEB yielded MAGs from orders Desulfobacterales, while HostZero and QIAamp enabled the assembly of genomes from Desulfarculales, Desulfobulbales, and Desulfovibrionales. Although all these orders are gram-negative bacteria, differences in their cell wall compositions may influence susceptibility to the enzymatic treatment used in pre-extraction host depletion kits [80, 81]. Similar observations were made for Campylobacterota (formerly “ε-proteobacteria”), specifically *Sulfurovum* sp. and *Arcobacter* sp. The depletion of microbial taxa that were possibly sensitive to the enzymatic treatment likely had less influence on MAG completeness than the reduction of host DNA itself. Comparable read mapping across assemblies from different treatments (Table 1), and reduction of host-associated reads (Fig. 3, Suppl. S8) support the notion that host depletion, rather than microbial loss, was the primary driver of improved genome recovery.

As an illustrative example of the advantage of using host depletion methods for metagenomic approaches, we assembled and further analyzed MAGs from the family Sedimenticolaceae (order Chromatiales) using QIAamp and HostZero (Fig. 4). Phylogenetic analyses placed these genomes in a distinct branch within the genus *Sedimenticola*, suggesting that they represent novel lineages. These free-living, sulfur-oxidizing bacteria are known associates of seagrass roots and likely contribute to plant health by detoxifying hydrogen sulfide [22]. Similar to *Candidatus* Thiodiazotropha [82, 83], another Sedimenticolaceae genus, members of *Sedimenticola*-like species have been identified as endosymbionts of the mangrove sediment clam species *Phacoides pectinatus* [84]. Whether these two Sedimenticolaceae genera also form endosymbiotic relationships with seagrasses remains to be investigated. Pre-extraction host depletion kits provide an effective means to study these potential symbioses by enabling detection of low-abundance sulfur-oxidizing bacteria that might otherwise be obscured by host and dominant symbiont DNA.

## Conclusion and outlook

This study is the first to systematically compare DNA extraction methods with and without host DNA depletion in seagrasses as examples of environmental samples. The post-extraction host depletion method NEB produced comparable microbial compositions and host contents as PowerSoil, however, also enabled the assembly of highly complete MAGs, which the control method did not achieve. In contrast, the pre-extraction host depletion methods QIAamp and HostZero resulted in distinct microbial community profiles and facilitated the recovery of a greater diversity of unique and potentially novel MAGs. While these methods introduce potential biases, they also reveal bacterial taxa that were otherwise undetectable with conventional protocols. Even though PowerSoil arguably carries its own established set of biases, and true detection of all taxa residing in environmental samples might still be obscured, we do not propose host depletion methods as a universal replacement, but rather as powerful complementary tools, especially valuable in high-diversity environments such as seagrass microbiomes. Nevertheless, further validation of these methods in other host-associated microbial communities will be necessary.

In conclusion, host DNA depletion methods enabled the detection of previously undetected taxa, and the modified protocols developed in this study hold strong potential for advancing future research into seagrass-associated microbial communities. Future work could apply these optimized protocols across diverse seagrass species or other marine plant holobionts to deepen our understanding of microbial contributions to plant health and ecosystem resilience.

## Supplementary Material

Supplementary_material_ycag082

## Data Availability

The genomic data generated in this study have been deposited at the Sequence Read Archive under the BioProject accession PRJNA1314029.
