# Supplementary material for: Host depletion kits improve microbiome analyses in environmental samples: seagrass as a test case
Source: ISME Commun. 2026 Mar 28;6(1):ycag082. doi: 10.1093/ismeco/ycag082 (PMC13236103; doi:10.1093/ismeco/ycag082)
Supplement: Supplementary_material_ycag082 [file supplementary_material_ycag082.zip › Supplementary_Protocols_ycag082.pdf]

Supplementary Protocols

Overview

Approximately 200 mg of root + rhizome sample was used for each extraction. DNA concentrations were measured using Qubit 4 (Thermo Fisher Scientific) with the Qubit dsDNA HS Assay Kit (Thermo Fisher Scientific, Cat. No. Q32851).

As a control, the DNeasy PowerSoil Pro Kit (Qiagen, Cat. No. 47014) was used to extract total DNA, including host and associated microbiota, according to the manufacturer's instructions. These samples served as a "baseline" for comparison with host DNA depletion kits.

Pre-extraction host depletion kits selectively lyse eukaryotic (host) cells and remove their DNA through DNase treatment. Modifications were made to both used protocols due to the liquid sample requirement. In the QIAamp DNA Microbiome Kit (Qiagen, Cat. No. 51704), after 30 min with end-over end rotation incubation in AHL buffer and centrifugation (first step in the protocol), the supernatant and pellet were separated and both retained. The supernatant was stored at 4°C, while the pellet was resuspended in 1 × Phosphate Buffered Saline (PBS; 1 × = 137 mM NaCl, 2.7 mM KCl, 10 mM Na_2HPO_4, 1.8 mM KH_2PO_4, pH 7.4), vortexed for 20 min, and filtered through a 40 µm cell strainer (Corning Falcon, Cat. No. 352340), then passed through a 5 µm cell strainer (pluriSelect, Cat. No. 43-10005-50). The filtrate and the reserved supernatant were combined and centrifuged at 10000 × g for 20 min before enzymatic treatment of the bacterial pellet according to manufacturer's instructions. In the HostZERO Microbial DNA Kit (Zymo Research, Cat. No. D4310), samples were resuspended in 1 ml 1 × PBS, vortexed for 20 min, and filtered through a 40 µm strainer. The filtrate was then passed through a 5 µm strainer and centrifuged at 10000 × g for 15 min prior to enzymatical lysis.

Post-extraction host depletion methods require high-quality DNA (long and unsheared fragments). For that reason, we extracted high-molecular-weight DNA using the Wizard HMW DNA Extraction Kit (Promega, Cat. No. A2920). The NEBNext Microbiome DNA Enrichment Kit (New England Biolabs, Cat. No. E2612S/L) was then used to selectively remove eukaryotic CpG-methylated DNA via magnetic beads. For both kits, manufacturer's instructions were followed.

PowerSoil (Qiagen, Cat. No. 47014) – minor modification

1. Add 800 µl of Buffer CD1 to the tube with the ground up sample. Vortex briefly to mix. Decant into the PowerBead Pro Tube. Vortex briefly to mix.
2. Secure the PowerBead Pro Tube horizontally on a Vortex Adapter for 1.5–2 ml tubes (cat. no. 13000-V1-24). Vortex at maximum speed for 20 min (if less than 12 preps at the same time, only for 10 min).
3. Centrifuge the PowerBead Pro Tube at 15,000 x g for 1 min.
4. Transfer the supernatant to a clean 2 ml Microcentrifuge Tube (provided; expect ~ 550 µl supernatant).
5. Add 200 µl of Solution CD2 and vortex for 5 s. Leave to incubate 5 min.
6. Centrifuge at 15,000 x g for 1 min at room temperature. Avoiding the pellet, transfer up to 700 µl of supernatant to a clean 2 ml Microcentrifuge Tube (provided).
7. Add 600 µl of Solution CD3 and vortex for 5 s. Leave to incubate 5min.
8. Load 650 µl of the lysate onto an MB Spin Column and centrifuge at 15,000 x g for 1 min.
9. Discard the flow-through and repeat step 8 to ensure that all of the lysate has passed through the MB Spin Column.
10. Carefully place the MB Spin Column into a clean 2 ml Collection Tube (provided). Avoid splashing any flow-through onto the MB Spin Column.
11. Add 500 µl of Solution EA to the MB Spin Column. Centrifuge at 15,000 x g for 1 min.
12. Discard the flow-through and place the MB Spin Column back into the same 2 ml Collection Tube.
13. Add 500 µl of Solution C5 to the MB Spin Column. Centrifuge at 15,000 x g for 1 min.
14. Discard the flow-through and place the MB Spin Column into a new 2 ml Collection Tube (provided).
15. Centrifuge at up to 16,000 x g for 2 min. Carefully place the MB Spin Column into a new 1.5 ml Elution Tube (provided).
16. Add (minimum of 30 µl) 50 µl of Solution C6 to the center of the white filter membrane. Leave to incubate 5 min.
17. Centrifuge at 15,000 x g for 1 min. Add the flowthrough again on top of the spin column. Leave to incubate 5 min.
18. Centrifuge at 15,000 x g for 1 min. Discard the MB Spin Column. The DNA is now ready for downstream applications.
19. Store DNA at -20°C.

QIAamp (Qiagen, Cat. No. 51704) – modification

1. Add 500 µl AHL buffer to the sample and incubate for 30 min at room temperature with end over end rotation.
2. Centrifuge the tube at 10,000 x g for 10 min and carefully remove the supernatant. Do not disturb the pellet as this will result in loss of bacterial material.
3. Remove the supernatant [1] and keep it on the side. Add 1ml 1 × PBS to the pellet and vortex 20 min.
4. Continue with pellet in PBS: filter through 40 µm cell strainer on a 50 ml Falcon tube (3234 x g, 1 min). Transfer ~ 500 µl of the filtered liquid on to a 5 µm filter on an 2 mL Eppendorf tube, 10000 x g, 1 min, and repeat with remaining liquid.
5. Add the kept supernatant [1] to the liquid and centrifuge for 20 min at 10000 x g.
6. Discard the supernatant, resuspend the pellet in 190 µl Buffer RDD and add 2.5 µl Benzonase. Mix well and incubate at 37°C for 30 min at 600 rpm in a heating block or water bath.
7. Add 20 µl Proteinase K and incubate at 56°C for 30 min at 600 rpm in a heating block or water bath.
8. Briefly spin the tube at slow speed to remove condensation. Add 200 µl Buffer ATL (containing Reagent DX). Mix well to avoid loss of sample material and transfer into a Pathogen Lysis Tube L.
9. Place the Pathogen Lysis Tube L into a FastPrep-24 instrument. Apply a velocity of 6.5 m/s twice for 45 s each with a 5 min intermission during which the samples should be stored on ice. After lysis, heat tubes for 5 min at 95°C.
10. Centrifuge the Pathogen Lysis Tube L at 10,000 x g for 1 min to reduce the amount of foam after lysis. Mix carefully and transfer the supernatant to a fresh microcentrifuge tube.
11. Add 40 µl Proteinase K, mix by vortexing, and incubate at 56°C for 30 min at 600 rpm in a heating block or water bath.
12. Add 200 µl Buffer APL2. Mix by pulse vortexing for 30 s. Incubate at 70°C for 10 min and briefly spin the tube.
13. Add 200 µl 100% ethanol to the lysate. Mix thoroughly by pulse vortexing for 15–30 s.
14. Carefully apply up to 700 µl of the mixture to the QIAamp UCP Mini Column without wetting the rim. Close the cap and centrifuge at 6,000 x g for 1 min.
15. Discard the flow-through. Put the column back into the collection tube to repeat with any remaining mixture.
16. Transfer the QIAamp UCP Mini Column to a fresh collection tube. Carefully open the cap and add 500 µl Buffer AW1 without wetting the rim. Close the cap and centrifuge at 6,000 x g for 1 min. Place the QIAamp UCP Mini Column into a fresh 2 ml collection tube and discard the filtrate.
17. Carefully open the QIAamp UCP Mini Column and add 500 µl Buffer AW2 without wetting the rim. Centrifuge at full speed (~ 20,000 x g) for 4 min.
18. Place the QIAamp UCP Mini Column into a fresh 2 ml collection tube. Discard the filtrate. Centrifuge at full speed (20,000 x g) for 2 min.
19. Place the QIAamp UCP Mini Column into a fresh 1.5 ml tube and apply 30-50 µl Buffer AVE directly onto the center of the membrane. Close the lid and incubate at room temperature for 5 min. Centrifuge at 6,000 x g for 1 min to elute the DNA. Store DNA at -20°C.

HostZero (Zymo Research, Cat. No. D4310) – modification

1. Resuspend sample in 1 ml 1 × PBS, vortex for 20 min and transfer the mixture onto a 40 µm strainer on a 50 mL falcon tube.
2. Centrifuge shortly (3234 x g, 1 min) to get the sample into the falcon. Transfer ~ 500 µl of the filtered liquid on a 5 µm filter on an 2 mL Eppendorf tube, short spin with centrifuge (10000 x g, 1 min), and repeat with remaining liquid.
3. Centrifuge the tube at 10,000 x g for 10 min. Without disturbing the pellet, carefully remove and discard the supernatant.
4. Add 100 µl of Microbial Selection Buffer to the tube and resuspend the pellet by pipetting up and down 10 times (depending on how difficult it is to resuspend).
5. Add 1 µl of Microbial Selection Enzyme to the suspension. Vortex briefly to mix.
6. Incubate the tube at 37°C for 30 min.
7. Add 20 µl of Proteinase K to the sample and vortex for at least 10 s. Incubate at 55°C for 10 min.
8. Add 100 µl of DNA/RNA Shield™ (2x Concentrate) to the sample and vortex for at least 10 s. Incubate at room temperature for 5 min.
9. Proceed to Microbial DNA isolation or store sample at -80°C.
10. Add the entire sample to a ZR BashingBead™ Lysis Tube (0.1 & 0.5 mm).
11. Add 750 µl of ZymoBIOMICS™ Lysis Solution to the tube and cap tightly.
12. Secure in a bead beater fitted with a 2 ml tube holder assembly and process 3 times at 6.5m/s for 1 min each, with breaks of 5 min in between. Store the samples on ice during the breaks.
13. Centrifuge at 10,000 x g for 1 min. Transfer the supernatant (expect about 400 µl) to a Collection Tube.
14. Add 1.2 ml of DNA Binding Buffer to the supernatant in the Collection Tube and mix by pipetting up and down 10 times
15. Transfer 650 µl of the mixture to a Zymo-Spin™ IC-Z Column4 in a Collection Tube and centrifuge at 10,000 x g for 1 min. Discard the flow-through.
16. Repeat step 15 twice, until the whole mixture has passed through the column.
17. Place the column in a new Collection Tube.
18. Add 400 µl of DNA Wash Buffer 1 to the column. Centrifuge at 10,000 x g for 1 min. Discard the flow-through.
19. Add 700 µl of DNA Wash Buffer 2 to the column and centrifuge at 10,000 x g for 1 min. Discard the flow-through.
20. Add 200 µl of DNA Wash Buffer 2 and centrifuge the column at 10,000 x g for 2 min. Discard the flow-through.
21. Transfer the column into a clean microcentrifuge tube.
22. Add 30 µl of DNase/RNase-Free Water directly to the column matrix. Incubate for 5 min. Centrifuge at 10,000 x g for 1 min.
23. Add the flowthrough again on top of the spin column. Leave to incubate 5 min. Centrifuge at 10,000 x g for 1 min. Store DNA at -20°C.

Wizard HMW (Promega, Cat. No. A2920) – Protocol for plant tissues – no modification

1. Process tissue by freezing with liquid nitrogen and grinding into a fine powder using a microcentrifuge tube pestle or a mortar and pestle. Add 200 mg of this powder to a 1.5 ml microcentrifuge tube.
2. Add 500 µl of HMW Lysis Buffer A, and vortex 1–5 s to wet the tissue.
3. Incubate at 65°C for 15–30 min.
4. Cool the lysate to room temperature for 5 min. Add 3 µl of RNase A Solution to the sample and mix by inverting the tube 5–7 times. Incubate the mixture at 37°C for 15 min.
5. Add 20 µl of Proteinase K Solution to each sample and mix the sample by inverting the tube 10 times. Incubate the mixture at 56°C for 15 min. Cool to room temperature for at least 5 min or chill on ice 1 min.
6. Centrifuge at 13,200 × g for 3 min at room temperature to pellet any insoluble material. Transfer the lysate to a clean 1.5 ml microcentrifuge tube.
7. Add 200 µl of Protein Precipitation Solution to the nuclear lysate. Using 1,000 µl wide bore pipette tips, mix the solution five times. Draw the tube contents from the bottom of the tube, then expel the lysate rapidly down the side of the tube. Small protein clumps may be visible after mixing. Incubate on ice for 5 min.

Note: If wide-bore pipette tips are unavailable, vortex lysate and Protein Precipitation Solution for 5 s. Do not tip mix.

8. Centrifuge at 14,100 × g for 10 min at room temperature. A greenish pellet should be visible. If any unpelleted debris is visible, repeat the centrifugation step.
9. Slowly transfer the supernatant to a clean 1.5 ml microcentrifuge tube by decanting the sample into a tube containing 600 µl of room-temperature 100% isopropanol.

Note: Some supernatant may remain in the original tube containing the protein pellet. Leave this residual liquid in the tube to avoid contaminating the DNA solution with the precipitated protein.

10. Gently mix the solution by gently inverting the tube eight times. Incubate for 1 min at room temperature and repeat the inversion. White thread-like strands of DNA may form a visible mass.
11. Centrifuge at 14,100 × g for 2 min at room temperature. The DNA may be visible as a small white pellet.
12. Decant the supernatant and add 600 µl of room temperature 70% ethanol to the DNA. Gently invert the tube several times to wash the DNA pellet and the sides of the microcentrifuge tube. Centrifuge as instructed in Step 11.
13. Discard the supernatant then repeat Step 12.
14. Carefully aspirate the ethanol. The DNA pellet is very loose at this point so carefully avoid disturbing or aspirating the pellet into the pipette. Invert the tube on clean absorbent paper and air-dry the pellet for 10–15 min.
15. Add 100 µl of DNA Rehydration Solution to the tube. Do not vortex because this will cause mechanical shearing and decrease average fragment size. Rehydrate the DNA by incubating the solution overnight at room temperature. Alternatively, incubate the purified DNA at 65°C for 1 hour, periodically mixing the solution by gently tapping the tube.
16. If DNA appears nonhomogeneous (e.g., undissolved pellet is still visible), mix with 200 µl wide-bore pipette tips. Store the DNA at 2–8°C.

NEBNext (New England Biolabs, Cat. No. E2612S/L) – no modification

Bead preparation:

1. Before proceeding, determine the bead volume necessary to enrich for microbial DNA:

   For every 6.25 ng of input DNA: 1 µl of MBD2-Fc-bound magnetic beads is needed for enrichment

2. Resuspend NEBNext Protein A Magnetic Beads by gently pipetting the slurry up and down until the suspension is homogeneous. Do not vortex.
3. Prepare 1 × Bind/wash Buffer on ice by diluting 1 part NEBNext Bind/wash Buffer (5 ×) with 4 parts DNase-free water. One individual reaction, from start to finish, will require 4 ml of 1 × Bind/wash Buffer.
4. In one tube, add 16 µl of MBD2-Fc protein and 160 µl of Protein A Magnetic Beads. For input amounts other than 1 µg, add (0.1 × Y) µl of MBD2-Fc protein and (Y) µl of Protein A magnetic beads. Mix by pipetting up and down until the beads are completely homogeneous, at least 5-10 times.
5. Mix the bead-protein mixture by placing the tube in a rotating mixer for 10 min at room temperature.
6. Briefly spin the tube and place on the magnetic rack for 2–5 min or until the beads have collected to the wall of the tube and the solution is clear.
7. Carefully remove the supernatant with a pipette without disturbing the beads.
8. Add 1 ml of 1 × Bind/wash Buffer (kept on ice) to the tube to wash the beads. Pipette up and down until the beads are completely homogeneous, at least 5-10 times.
9. Mix the beads on a rotating mixer for 3 min at room temperature.
10. Briefly spin the tube and place on the magnetic rack for 2–5 min or until the beads have collected to the wall of the tube and the solution is clear.
11. Carefully remove the supernatant with a pipette without disturbing the beads. Repeat steps 8–11.
12. Remove the tube from the rack and add 160 µl of 1 × Bind/wash Buffer (kept on ice) to resuspend the beads. For input amounts other than 1 µg, add (Y) µl of 1 × Bind/wash Buffer to resuspend the beads. Mix by pipetting up and down a few times.
13. The MBD2-Fc-bound magnetic beads are stable for up to 7 days at 4°C.

Methylated host DNA:

14. Add 1 µg in up to 200 µl input DNA to the tube containing the 160 µl of MBD2-Fc-bound magnetic beads. For other DNA input amounts, add DNA to (Y) µl of MBD2-Fc bound magnetic beads.
15. Add undiluted Bind/wash Buffer (5 ×) for a final concentration of 1 ×. (for example add 10 µl of Bind/wash Buffer (5 ×) if the DNA input sample was 40 µl, add 4 µl of Bind/wash Buffer (5 ×) if the DNA sample was 16 µl). Pipette the sample up and down until the beads are completely homogenous, at least 5-10 times.
16. Agitate the tube on a rotating mixer for 15 min at room temperature with rotation.

Collect Enriched microbial DNA:

17. Briefly spin the tube and place on the magnetic rack for 5 min until the beads have collected to the wall of the tube and the solution is clear.
18. Carefully remove the supernatant with a pipette, without disturbing the beads and transfer it to a clean microcentrifuge tube. This supernatant contains the target microbial DNA. Store this sample at –20°C or proceed directly to purification:

Ethanol Precipitation:

19. Add 2.5 volumes of 100% ethanol, incubate for 10 min on ice, then centrifuge the sample for 30 min at 13,000 rpm (16,000 rcf). Remove the ethanol, allow the pellet to air dry, and then resuspend the pellet in a small quantity (50 µl) of 1 × Tris-EDTA (TE; 1 × = 10 mM Tris-Cl, 1 mM EDTA, pH 8.0) buffer.
20. If the ethanol pellet contained any residual beads, the resuspended sample can be placed on the magnetic rack 5 min to concentrate the beads on the inner wall of the tube, and the supernatant can be transferred to a fresh microcentrifuge tube.
